# Adhesion G protein-coupled receptor, ELTD1, is a potential therapeutic target for retinoblastoma migration and invasion

**DOI:** 10.1186/s12885-020-07768-3

**Published:** 2021-01-11

**Authors:** Jonathan Guihurt Santiago, Neikelyn Burgos-Tirado, Daniella Dorta Lafontaine, José C. Mendoza Sierra, Roberto Herrera Camacho, Clara M. Vecchini Rodríguez, Vanessa Morales-Tirado, Jacqueline Flores-Otero

**Affiliations:** 1 Institute of Neurobiology, University of Puerto Rico, Medical Sciences Campus, San Juan, Puerto Rico; 2 University of Puerto Rico, Rio Piedras Campus, Rio Piedras, Puerto Rico; 3 Present address: Debusk College of Osteopathic Medicine at Lincoln Memorial University, Harrogate, TN USA; 4 Present address: Department of Pharmacology, University of Michigan Medical School, Ann Arbor, MI USA; 5 Present address: Central University of the Caribbean of Puerto Rico, Bayamon, Puerto Rico; 6 University of Medicine and Health Sciences, New York, USA; 7 Current affiliation: Ponce Health Sciences University, Ponce, Puerto Rico; 8 Department of Anatomy and Neurobiology, University of Puerto Rico, Medical Sciences Campus, PO Box 365067, San Juan, 00936-5067 Puerto Rico; 9 University of Puerto Rico Comprehensive Cancer Center, San Juan, Puerto Rico; 10 Department of Ophthalmology, Hamilton Eye Institute, University of Tennessee Health Science Center, Memphis, TN USA; 11 Department of Microbiology, Immunology and Biochemistry, University of Tennessee Health Science Center, Memphis, TN USA; 12 Present address: AbbVie Bioresearch Center, Worcester, MA USA

**Keywords:** Retinoblastoma, Adhesion-GPCRs, ELTD1, GPR125, Intraocular cancer

## Abstract

**Background:**

Prognosis for pediatric metastatic Retinoblastoma (Rb) is poor and current therapies are limited by high systemic toxicity rates and insufficient therapeutic efficacy for metastatic Rb. Tumor dissemination to the brain is promoted by the heterogeneous adhesive and invasive properties of Rb cells within the tumor. In this study we evaluate, for the first time, the expression, and roles of the ELTD1 and GPR125 adhesion G protein-coupled receptors (GPCRs) in Rb cell migration, viability and invasion.

**Methods:**

We characterized the RNA expression of adhesion-GPCRs in 64 Rb tumors compared to 11 fetal retinas using the database from the Childhood Solid Tumor Network from St Jude Children’s Research Hospital. The role of ELTD1 and GPR125 in Rb were investigated ex vivo by microarray analysis, in vitro by cell viability, Western blot and migration assays, in addition to imaging of the subcellular localization of the GPCRs. To elucidate their role in vivo we utilized siRNA technology in an established Rb orthotopic xenograft murine model.

**Results:**

Our investigation demonstrates, for the first time, that ELTD1 but not GPR125, is significantly increased in Rb tumors compared to fetal retinas. We utilized established the Rb cell lines Y79 and Weri-Rb-1, which represent an aggressive, metastatic, and non-metastatic phenotype, respectively, for the in vitro analyses. The studies demonstrated that ELTD1 is enriched in Weri-Rb-1 cells, while GPR125 is enriched in Y79 cells. The measured differences extended to their subcellular localization as ELTD1 labeling displayed punctate clusters in cell-to-cell adhesion sites of Weri-Rb-1 cells, while GPR125 displayed a polarized distribution in Y79 cells. Lastly, we demonstrated the lack of both adhesion receptors does not affect Rb cell viability, yet inhibition of ELTD1 decreases Y79 cell migration in vitro and invasion in vivo.

**Conclusion:**

Taken together, our data suggest that ELTD1, is a potential target to prevent extraocular Rb. The results within establish ELTD1 as a potential therapeutic target for metastatic Rb.

**Supplementary Information:**

The online version contains supplementary material available at 10.1186/s12885-020-07768-3.

## Background

Retinoblastoma (Rb) is the most common intraocular tumor that threatens pediatric survival rates if not diagnosed and treated early. Upon mutation of both alleles of the tumor suppressor *RB1* gene, cells from the retina abnormally proliferate to create a tumor mass that disrupts intraocular structures. The dissemination of the disease leads to central nervous system (CNS) metastasis, increasing mortality rates. Current therapies aim to limit tumor proliferation and growth. The administration of these therapies remains challenging due to several clinical factors, including patient’s age, sex, laterality, vascularity, staging at diagnosis [2, 6, 11, 12, 32], and tumor heterogeneity [8, 17]. Recent work has shown Rb cell heterogeneity influences resistance to therapies as some tumor cells display different degrees of invasiveness and aggressiveness within the same tumor mass [35]. Therefore, this highlights the importance of identifying novel therapeutic modalities that target Rb cells with varied metastatic potential.

Adhesion G protein-coupled receptors (adhesion-GPCRs) have been at the forefront of cancer research for their roles in [3, 20, 40], regulating cellular adhesion, polarity, migration and angiogenesis [21, 27]. To date, 33 receptors have been identified, with at least 15 of these being dysregulated in distinct tumor types [3]. To our knowledge, no prior study has elucidated the role of adhesion-GPCRs in Rb. We focused our investigation on two of these adhesion-GPCRs in Rb tumors, the epidermal growth factor, latrophilin and seven transmembrane domain containing 1 (ELTD1/ADGRL4) and the G-protein receptor 125 (GPR125/ADRGRA3). While ELTD1 is associated with glioblastoma, colorectal cancer, cardiac and renal function, [1, 14, 26, 33, 34, 39, 42], GPR125 plays vital roles during embryonic development, cell adhesion, signaling and planar cell polarity [19]. Overexpression of GPR125 has also been reported after brain injury and myeloid sarcoma formation, while its downregulation during colorectal cancer is a predictive biomarker of poor prognosis and higher probability of recurrence [13, 29, 38]. Our aim was to investigate the role of these two adhesion-GPCRs in Rb, as both ELTD1 and GPR125 influence multiple aspects of cancer during disease development and progression. To accomplish this, we utilized a combination of in vivo work, ex vivo tumors and the established and well-characterized Rb cell lines, Y79 and Weri-Rb-1 cells, representing the metastatic and non-metastatic phenotype. Our results demonstrate that ELTD1is a potential target to prevent Rb cell migration and optic nerve dissemination, while GPR125 may play a role in Rb cell adhesion.

## Methods

### Cell culture

Y79, Weri-Rb-1 cells (ATCC-HTB-18 and ATCC-HTB-169, respectively), and HEK-293 (ATCC-CRL-1573) cells were obtained from the American Type Culture Collection (ATCC, Manassas, VA) biological resource center [23, 31]. Rb355 Rb cells were kindly provided by the Childhood Solid Tumor Network (CSTN) at St Jude Children’s Research Hospital (SJCRH) [36]. All Rb cell lines were cultured as previously described [37]. HEK-293 cells were maintained in complete growth Dulbecco’s modified Eagle’s medium (DMEM; Sigma Aldrich, 51435C), supplemented with 10% FBS, 0.1% penicillin–streptomycin and 2 mM glutamine (Pen/Strep, Fisher Scientific, BP295950; 2 mM Glutamine, Fisher Scientific 25030081). These were expanded every 2 to 4 days after they reached 70 to 80% confluence. Jurkat cells were kindly provided by Dr. Guillermo A. Yudowski (Institute of Neurobiology, San Juan, PR).

### MicroArray

Adhesion-GPCR microarray expression data were obtained from the CSTN at SJCRH [36]. This dataset of genes was analyzed from 64 human Rb tumors compared to 11 fetal retinas using the JMP v12.2 Software (www.jmp.com/en_us/software/jmp.html) to conduct a hierarchical cluster analysis (HCA). Ward’s method, in which clustering is based on the within-cluster sum of square, was applied for the HCA and dendrogram, and supervised heat maps were presented. Boxplots were generated to graphically examine the distribution of log base 2 transformed data of expression per select gene per group (normal retina vs. retinoblastoma) and a series of ANOVA tests were conducted to determine the group difference of expression level per select gene using SAS 9.3 (www.sas.com/en_us/software/analytics/stat.html).

### RT-PCR

#### RNA isolation and cDNA synthesis

Sections of paraffin-embedded enucleated eyes from Rb patients were cut approximately 8 μm thick before being placed in a sterile RNase free microcentrifuge tube (MidSci, St. Louis, MO). Each sample was incubated with deparaffinization solution (Qiagen, Valencia, CA) and incubated at 56 °C for 3 mins followed by Proteinase K incubation. RNA isolation was performed using the Qiagen® miRNease Mini Kit (Qiagen) using manufacturer’s specifications. RNA concentration was assessed using a Nanodrop Spectrophotometer (Nanodrop, Wilmington DE). A total of 50 ng of RNA was used to synthesize cDNA using the SuperScript® VILO™ cDNA Synthesis Kit (Life Technologies, Carlsbad, CA).

#### cDNA pre-amplification

Material was pre-amplified using cDNA, TaqMan® PreAmp Master Mix and a cocktail of the pooled primers mix listed in Table 1. Enzyme activation was carried out at 95 °C for 10 mins, followed by 14 cycles of amplification (95 °C for 15 s, followed by 60 °C for 4 mins). Pre- amplified material was diluted 1:10 in Tris EDTA buffer and kept at − 20 °C until ready for use. Gene targets were previously tested using an amplification efficiency test.

**Table 1:** List of primers utilized in the RT-PCR studies

Gene	Species	Company	Assay Number
*HPRT1*	Human	Life Technologies	Hs02800695_m1
*GPR125*	Human	Life Technologies	Hs00402930_m1
*ELTD1*	Human	Life Technologies	Hs00223377_m1

#### qPCR reaction

Each reaction was prepared in a 10uL final volume, containing the TaqMan Universal Master Mix, the diluted cDNA, primers, and nuclease free water, as previously described [9, 10]. Samples were analyzed using the Roche LightCycler® 480 using the following conditions: hold step of 50 °C for 2 mins followed by 95 °C for 10 mins. Thermal cycling was programmed to run 40 cycles of 95 °C for 15 s followed by 60 °C for 1 min. All measurements were done in replicates of 4. Relative quantitation was performed using the Comparative C_T_ Method using the values of the endogenous or housekeeping gene (*HPRT1*) and target genes in each sample (Table 1). The relative fold change was calculated using the following equation: *R*_q_ = 2^−ΔC^_T_, where ΔC_T_ = C_T_ target gene − C_T_ reference gene. Data are presented as mean ± SEM. Differences were assessed by Prism, Graph Pad (Graph Pad, La Jolla, CA).

### Fetal retina and retinoblastoma human samples

Human fetal retina was purchased from Advanced Bioscience Resources (ABR, Alameda, California). Upon arrival, retina tissue was dissected out of the eyecup, cut into small pieces, placed in sterile Eppendorf tubes and flash frozen in dry ice prior to their storage at a − 80 °C until ready to use for protein extraction and Western blot assays. Rb tumor samples were obtained from the CSTN at SJCRH. All studies received approval from the University of Puerto Rico Medical Science Campus Institutional Review Board (IRB) (Approval #B0580215).

### Western blot assay

Cell cultures of Y79, Weri-Rb1, Rb355, HEK-293 and Jurkat cells were incubated for 48–72 h before they were harvested, lysed, and quantified for Western blot analysis. Human brain and HeLa lysates were both obtained from Novus Biologicals, LLC (Centenial, CO; Cat #NB820–59177 and NB800-PC1, respectively). Cells were lysed for 5 min using cold Radioimmunoprecipitation assay (RIPA) buffer (Sigma, R0278) containing phosphatase and protease inhibitors (cOmplete™ Protease Inhibitor Cocktail, Sigma Aldrich, 11–836–145-001) and centrifuged at 12,000 rpm for 10 min. Retinoblastoma (Rb) tumor samples were derived from human tumors established in orthotopic xenograft mouse model as previously described [24, 36]. Rb tumors stored at − 80 °C were lysed for 5 min using cold RIPA buffer containing phosphatase and protease inhibitors, sonicated at 10% maximum amplitude for a run time of 3 s, constant sonication, and centrifuged at similar condition as the fetal retina (control), the HEK-293 and Jurkat cells. Total protein extracts were collected from the supernatant. Protein concentration was measured using BCA Protein Assay (Thermo Scientific, 23,227) and a NanoDrop 2000 Spectrophotometer. Approximately 25μg-30μg of heated protein samples were electrophoresed using 4–20% pre-cast gels (BioRad, 456–1094) followed by transfer to Polyvinylidene fluoride (PVDF) membranes (Millipore, USA). To assess quality of protein transfer, membranes were stained with Ponceau S solution (Sigma, P7170), followed by rinsing 3x with Tris buffered saline with Tween 20 (TBS-T) for 5 min each. PVDF membranes were blocked with 5% Bovine Serum Albumin (BSA) dissolved in TBS containing 0.05% Tween-20 for 1 h at room temperature. After the blocking step, membranes were incubated with polyclonal goat anti-human GPR125 (LifeSpan BioSciences, B9196; 1:250, overnight at 4 °C) or with polyclonal rabbit anti-human ELTD1 (LS Bio., LS-C379622, 1:500, overnight at 4 °C) primary antibodies. Monoclonal mouse anti-GAPDH was used as a loading control (Santa Cruz Biotech, SC-32233, 1:500, overnight at 4 °C). Precision Plus Protein Dual Color Standards (BioRad 161–0374) were used to estimate molecular weight of proteins examined. After rinsing with TBS-T three times for 5 min, membranes were incubated for 1 h at room temperature with rabbit anti-goat IgG (Everest Biotech, EB2ND-001-Horseradish Peroxidase: HRP, 1:500, for GPR125), goat anti-rabbit HRP (Thermo scientific 32,460, 1:500, for ELTD1) or goat anti-mouse HRP (Thermo Scientific, 32,430, 1:500, for GAPDH) secondary antibodies conjugated to horseradish peroxidase. All antibodies were diluted in 2.5% BSA containing 10% Tween-20 and 0.01% Sodium Dodecyl Sulfate (SDS). Washing was completed with TBS-T for 5 min followed by TBS 2x for 5 min. Lastly, membranes were incubated with SuperSignal West Femto Substrate (Thermo Scientific, 34,096) for 5 min and immunodetection was obtained by using a chemiluminescent Kodak Image Station. Relative protein expression in blot images was analyzed with Image Studio Lite Version software (version 5.2.5).

### Indirect immunofluorescence assay

Y79 and Weri-Rb-1 cells were cultured into 35 mm petri dishes that contained 10% Poly-L Lysine (Sigma P4832) coated glass coverslips. After seeding cells at a density of 1.0 × 10^6^/mL in coated plates for 30 min, they were fixed with 4% paraformaldehyde (PFA: EMS 13710) for 2 h at 4 °C. After three rinses with 1x Phosphate Buffered Saline (PBS: 5 min/rinse), cells were blocked with 5% BSA (Sigma A9647) for 1 h at room temperature. Primary antibodies (diluted in 2.5% BSA) rabbit polyclonal anti-ELTD1 (LS Bio., LS-C379622; 1:500) or rabbit polyclonal anti-GPR125 antibodies (Abcam; ab51705; 1:500) were applied to cells and these were incubated overnight at 4 °C. Staining was then followed with anti-rabbit Alexa Fluor 488 secondary antibody (Life Technologies A21206; 1:1000 diluted in 2.5%) for 30 min at room temperature. Before direct double staining with a 0.4% monoclonal Texas-Red-conjugated Phalloidin antibody (Molecular Probes T7471), cells were blocked with 5% BSA for 30 min. In between steps, cells were rinsed three times for 5 min with 1X PBS. Mounting media containing 4′,6-Diamidino-2-Phenylindole dihydrochloride (DAPI: Life Technologies P36962) was applied prior to imaging.

### Cell transfection

Y79 and Weri-Rb-1 cells were transfected using the Qiagen’s manufacturer’s protocol. Briefly, 2 × 10^5^ cells/well were seeded in 24-well plate. Cells were either non-transfected (controls) or transfected with 100 nM si-ELTD1 (Qiagen, FlexiTube GeneSolution GS64123 for ELTD1, Cat. No. GS64123, Product No. 1027416) or si-GPR125 (Qiagen, FlexiTube GeneSolution GS166647 for GPR125, Cat. No. GS166647, Product No. 1027416). HiPerfect Transfection Reagent (Qiagen, Cat No./ID: 301707) was used to improve transfection efficiency and, after 48 h, viability and transwell assays were conducted.

### Cell viability assay

Transfected cells and respective non-transfected controls were assayed for viability using Trypan Blue. Cells were seeded in 12-well plates containing RPMI with 10% FBS and incubated at 37 °C for 24 h. After cell collection, these were diluted in Trypan Blue Dye (1:1 dilution) and quantified for viability with a hemocytometer. Viable cells remain unlabeled while non-viable cells were labeled blue due to compromising of the cellular membrane. Percentage of cell viability was determined using a Nexcelom Cellometer 2000 Cell Counter System.

### Transwell assay

Cell migration was conducted by using Boyden chamber assays to examine Rb cell migration. These contained 8 μm-pore-size polyethylene terephthalate basement membranes (BM) with a Falcon cell-culture insert (BD Biosciences, Bedford, MA). Y79 and Weri-Rb-1 cells (2 × 10^5^ cells/well) were seeded in 100uL serum-starved media (RPMI without serum) into the upper chamber, while 500uL of RPMI supplemented media with 10% FBS was added to the lower chamber. Seeding conditions of Rb cells (Y79 and Weri-Rb-1) were as follows: non-transfected cells, negative control (non-targeted RNA), and either *siELTD1* or *siGPR125*. Once the cells were seeded, the membrane was inserted into the lower chamber and cells were incubated for 24 h at 37 °C. Following the incubation period, the non-migrating cells were removed from the upper chamber by gently scraping with a cotton swab moistened with 1x PBS 5 times. Fixation and staining of cells located on the lower side of the insert membrane was achieved using cold methanol (10–15 min) and 1:10,000 Hoechst dye (Sigma, B2261) at room temperature for 30 min. Cells were mounted using Richard-Allan Scientific™ Cytoseal™ 60 mounting media (ThermoScientific, 8319–16). Images of four different fields were taken using a 20x objective and cells were counted to calculate the average number of cells that migrated through the membrane. To ensure that quantified Rb cells corresponded to migrating Hoechst-labeled cells, confocal Z stack images were taken for 3D reconstruction and quantification as validated by a maximum intensity projection image of each membrane (20x; Nikon Inverted Microscope Model TI, with Confocal, along with a Nikon Imaging Software-Confocal).

### Orthotopic xenograft Rb mouse model

Y79 and Weri-Rb-1 cell lines are well established and were previously demonstrated to follow metastatic and non-metastatic patterns in vivo, respectively [8]. We utilized these cell lines to do the orthotopic xenograft Rb murine model [17, 25]. Prior to eye injections or intraocular pressure measurements, mice were anesthesized with 2% isoflurane via gas (air) delivery of 2 L/min. Our in vivo conditions included a total of 5 control mice (inoculated with untransfected Y79 cells) as well as a total of 7 experimental mice (inoculated with Y79 cells that were previously transfected with *siELTD1*). Cells were inoculated into the vitreous of ICR-SCID mice (Taconic Laboratories, ICRSC-M, IcrTac:ICR-Prkdc^scid^). Approximately 50,000 cells were injected in a 5 μL final volume per eye into anesthetized ICR-SCID (IcrTac:ICR-Prkdc^scid)^ mice. The intraocular pressure (IOP) of inoculated mice was measured using a tonometer on a weekly basis as a signature of tumor growth. Mice with IOP ≥ 15 were euthanized in a CO2 chamber, followed by cervical dislocation and lack of withdraw reflex to assure animal death. Eyes were carefully dissected to preserve the attached optic nerve (ON). Dissected eyes were fixed in 4% PFA overnight at 4 °C and subsequently embedded in paraffin. Rb tumor invasion to the ON was analyzed with histological sections of inoculated eyes and extensive invasion was defined when tumor was detected ~ 200-300 μm beyond the ON head. Eyes embedded in paraffin were sectioned (5 μm) in the sagittal plane through the ON and stained with H&E. Tumor invasion to the anterior chamber, cornea, ciliary epithelium, retina and optic nerves were compared across transfected and non-transfected eyes. These animal studies were conducted in accordance with the University of Puerto Rico (UPR) Institutional Animal Care and Use committee (IACUC) guidelines (IACUC #A190115).

### Microscopy

Membranes of the transwell assays as well as ELTD1 and GPR125 protein distribution in Rb cells were imaged using a Nikon Inverted Microscope Model TI, with Confocal, along with a Nikon Imaging Software-Confocal. A 20x objective (Numeric Aperture: 0.5, Camera type Andor DU-897) was used to visualize and quantify migrating Hoechst-labeled Rb cells, while 20x and 40x objectives were used to examine ELTD1 and GPR125 protein distribution. In vivo H&E labeling was imaged using a Fluorescent Nikon Eclipse Ts2 microscope using 4x and 10x objectives. Imaging settings were the same across evaluations, according to each respective assay.

### Statistical analysis

All data analysis was done using GraphPad PRISM 8. Results were examined by Ordinary One-Way ANOVA data analysis of variance followed by a Tukey’s multiple comparison test or by a student’s paired T test. Significance of these tests were considered when *p < 0.05, **p < 0.01, ***p < 0.001 and ****p < 0.0001.

## Results

### RB tumors expressed high levels of *ELTD1*, but not *GPR125*, RNA

**Fig. 1:**
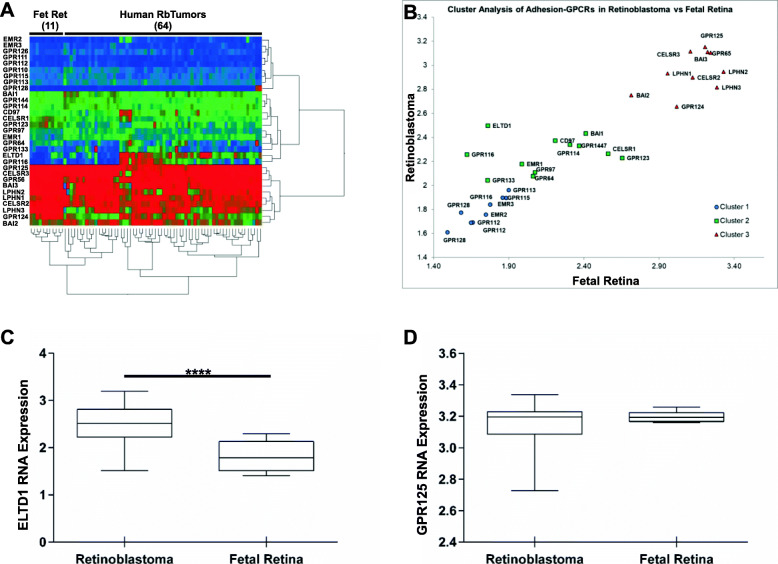
Adhesion G protein-coupled receptors (adhesion-GPCR) gene microarray profiling in human retinoblastomas (Rb). **a** Expression data from the Childhood Solid Tumor Network at St Jude Children’s Research Hospital containing the RNA expression of 31 members of the adhesion-GPCR orphan receptors were analyzed in 11 fetal retinae compared to 64 Rb tumor samples. **b** Hierarchical cluster analysis show three clusters of adhesion-GPCRs in normal fetal retinae and Rb tissue samples. **c**, **d** Box and whiskers plots display significant upregulation of *ELTD1* in Rb tumors compared to fetal retinae, (*p* < 0.0001) but no difference is detected in the RNA expression of *GPR125* (*p* = 0.1731)

An RNA profile comparing adhesion GPCRs was generated from a cohort of 64 human pediatric retinoblastoma tumor tissues and 11 human control fetal retinas. As shown in Fig. 1a, the heatmap of 31 adhesion-GPCRs showed distinct RNA expression patterns of some, but not all receptors in Rb tumors compared to fetal retina controls (Additional file 1: Supplementary Table 1). Cluster analyses reflected three distinct groups of adhesion-GPCRs expressed in the Rb tumors relative to fetal retina (Fig. 1b). Quantitative statistical analysis of the RNA expression signatures of these receptors showed that while *ELTD1, EMR1, EMR2, CD97* and *GPR97* were upregulated in Rb tumors, *LPHN2, LPHN3, GPR124, CELRS2* and *GPR56* were downregulated (Additional file 1: Supplementary Table 1). *LPHN1, EMR2, GPR125, GPR110, GPR111, GPR115, BAI1, BAI2, BAI3, GPR64, GPR112, GPR114, GPR128, CELSR3* and *GPR144* were not found to display any difference between tumor and normal fetal retina. For the current study we focused on two adhesion-GPCRs, ELTD1 and GPR125, both which influence different aspects of cancer development and progression [1, 13, 26, 38]. As shown in Fig. 1c-d, *ELTD1*, but not *GPR125* RNA expression, was significantly increased in Rb tumors compared to fetal retina controls (ELTD1: *p* < 0.0001 versus GPR125: *p* = 0.1731).

### ELTD1 protein was upregulated in RB cells and tumors

**Fig. 2:**
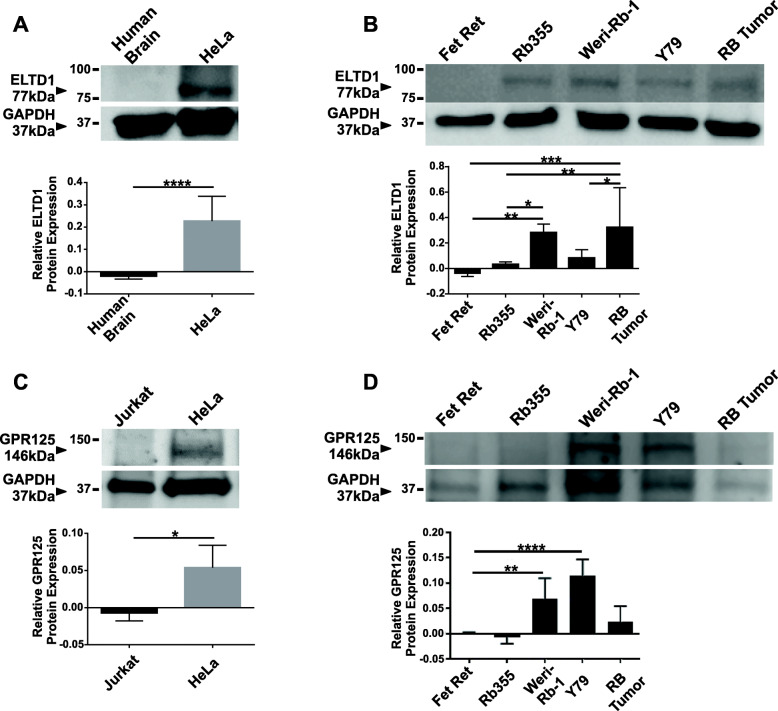
Western blot analysis show overexpression of ELTD1, but not GPR125, in Rb human tumors compared to fetal retina samples. **a**, **c** Immunoblot illustrate specificity of ELTD1 (77 kDa) and GPR125 (146 kDa) antibodies, respectively. For ELTD1 antibody specificity, human brain and HeLa cell lysates were used as negative and positive controls, respectively. For GPR125 antibody specificity, Jurkat and HeLa cell lysates were used as negative and positive controls, respectively. **b** Quantitative analysis show a significantly higher ELTD1 protein expression in Rb cells as well as in Rb tumors compared to fetal retina controls. **d** Quantitative analysis show a significantly higher GPR125 protein expression in Rb cells, Weri-Rb-1 and Y79, but not in Rb tumors compared to fetal retina controls. GAPDH (37 kDa) antibody was utilized as a loading control. Images shown are cropped from full-length blots presented in Additional File 2: Supplementary Figure 1, to improve the clarity and conciseness of the data presented. Relative protein expression in blot images was analyzed with Image Studio Lite Version software (version 5.2.5). Two-tailed Student’s t test (A and C) and Ordinary One-Way ANOVA followed by Tukey’s multiple comparison test (B and D) were used to compare each column with their control counterpart. Error bars, SEM. Significance was expressed as: * *p* < 0.05, **p < 0.01, ***p < 0.001 and ****p < 0.0001

Next, we investigated the protein levels of ELTD1 and GPR125 in Rb tumors and Rb cell lines by immunoblotting (Fig. 2). The ELTD1 antibody specificity was tested using human brain lysates as negative controls and HeLa lysates as positive controls (Fig. 2a; ****p < 0.0001; *N* = 10). Given our RNA microarray results, we hypothesized ELTD1, but not GPR125, to be increased in Rb cells and tumors. As illustrated in Fig. 2b, ELTD1 showed high levels in Weri-Rb-1 cells and Rb tumors compared to fetal retina (Fetal retina versus Weri-Rb-1 cells: **p < 0.01; Fetal retina versus Rb tumors: ***p < 0.001; *N* = 8), but this significant difference did not extend to the aggressive Y79 cells. The GPR125 antibody specificity was tested using Jurkat cell lysates as negative controls and HeLa lysates as positive controls (Fig. 2c; *p < 0.05; *N* = 6). We hypothesized there would be no difference in GPR125 protein expression based on the RNA microarray results which compared fetal retinas with human Rb tumors (Fig. 1). As expected, the GPR125 protein expression was not different between fetal retina and Rb tumors. However, Weri-Rb-1 and Y79 cells displayed a significant increase in GPR125 protein expression when compared to fetal retina samples Fig. 2d; (Fetal retina versus Weri-Rb-1: **p < 0.01 and Fetal retina versus Y79: ****p < 0.0001; *N* = 6).

### ELTD1 was enriched in cell-to-cell adhesion sites of the non-metastatic, Weri-Rb-1, cells, while GPR125 was distributed in a polarized manner in Y79, cells

**Fig. 3:**
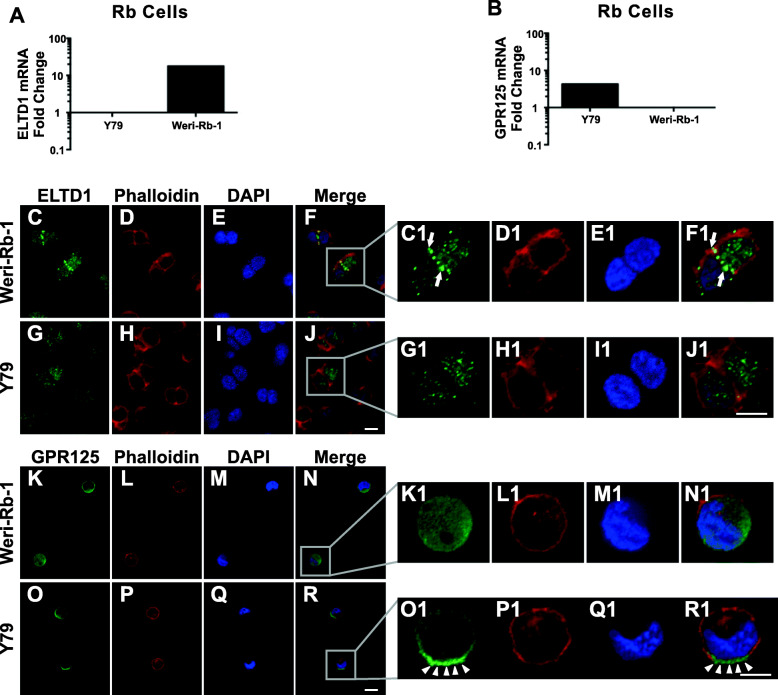
RT-PCR and immunocytochemistry analysis show distinctive mRNA and protein expression in Weri-Rb-1 and Y79 cells. **A-B.** *ELTD1* (A) and *GPR125* (B) mRNA expression in Y79 and Weri-Rb-1 cell lines. Results expressed as relative fold change of target gene over *HPRT1* as control. **C-J.** Low magnification images of anti-ELTD1 (green) antibody labeling show punctate clusters in cell to cell adhesion sites mostly prominent in the more adhesive Weri-Rb-1 cells. **C1-J1**. High magnification images of insets in panels F and J emphasizing the highly intense ELTD1 clusters in cell to cell adhesion sites (white arrows; Weri-Rb-1 cells), cytoplasm and nucleus of both Rb cell lines. **K-R.** Low magnification images of anti-GPR125 (green) antibody labeling show uniform labeling in the nucleus and cytoplasm of the less invasive Weri-Rb1 cells, while in the more invasive Y79 cells it showed enriched labeling in presumptive leading edge sites (white arrowheads). **K1-R1.** High magnification images of insets in panels N and R emphasizing the highly intense GPR125 labeling in presumptive leading edges of Y79 cells. **D, H, D1, H1, L, P, L1, P1**. Anti-Texas-Red conjugated Phalloidin antibody labeling of F-actin fibers in the Weri-Rb1 and Y79 cells. **E, I, E1, I1, M, Q, M1, Q1.** DAPI labeled nuclei of Weri-Rb-1 and Y79 cells. **F, J, F1, J1, N, R, N1, R1**. Merged images which were equally adjusted to improve representation. Scale bar for panels C-J and K-R is 10 μm and for panels C1-J1 and K1-R1 is 5 μm

To address whether *ELTD1* and *GPR125* mRNA are differently expressed in the Y79 and Weri-Rb-1 cells, we conducted RT-PCR analysis, as shown in Fig. 3A-B. The results demonstrated a differential expression of the tested adhesion-GPCRs in the Rb cell lines. The differential expression was both at the transcriptional (Fig. 2b) and protein (Fig. 2d) levels. Next, we investigated the subcellular distribution of these adhesion-GPCRs (Fig. 3C – R1). The intensity of ELTD1 labeling was more abundant at cell-to-cell adhesion sites in Weri-Rb-1 cells (Fig. 3C and C1; white arrows) compared to Y79 (Fig. 3G and G1), where ELTD1 displayed a punctate labeling pattern in the cytoplasmic and nuclear regions. The labeling profile of GPR125 was dispersed, not punctuated. Weri-Rb-1 cells showed a diffused profile (Fig. 3K, K1), while Y79 displayed a polarized GPR125 labeling (Fig. 3O and O1; white arrowheads).

### Silencing of *ELTD1* decreased migration, but not viability of the metastatic, Y79, cells

**Fig. 4:**
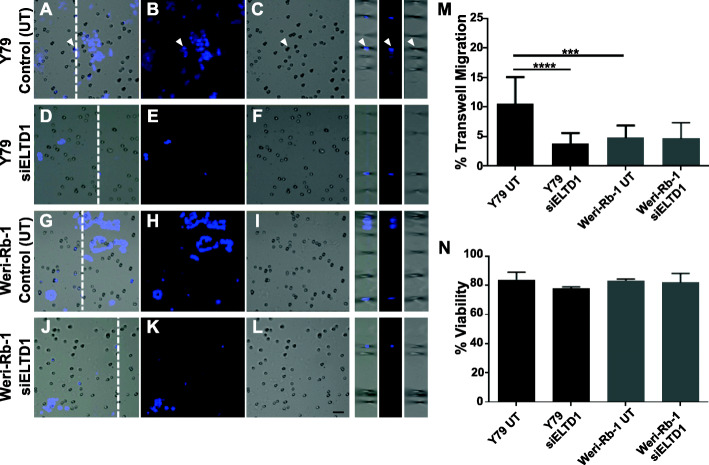
ELTD1 Silencing significantly reduced cell migration in Y79 Rb cells. **a-l** Rb cell lines were transfected with HiPerfect Reagent to induce silencing of *ELTD1*. Control untransfected (UT) cells (Y79: A-C; Weri-Rb-1: G-I), *siELTD1* transfected Y79 (D-F) and *siELTD1* transfected Weri-Rb-1 cells (J-L) were cultured in serum free media in the upper chamber of an 8 um polycarbonate membrane to measure cell migration. Cells were incubated for 6 h to enable migration to the bottom chamber by provision of complete media. Migrating cells were fixed and labeled with Hoechst dye to enable quantification of migrating cells (white arrowhead). **m** Quantitative analysis show a significant reduction in migration of *siELTD1* cells compared to UT cells. This effect was specific to Y79 cells, as Weri-Rb- 1 cell migration was unaffected. **n** Silencing of *ELTD1* did not have an effect in Rb cell viability. Ordinary One-Way ANOVA followed by Tukey’s multiple comparison test (M and N) was used to compare between columns. Scale bar for panels A-L is 10 μm. Error bars, SEM. Significance was expressed as ***p < 0.001 and ****p < 0.0001

To determine the functional effects of ELTD1 in Rb, we modulated gene expression by RNA silencing. Given the RNA upregulation of *ELTD1* in Rb tumors (Fig. 1) we hypothesized that silencing of *ELTD1* would reduce Rb cell migration. In addition, we hypothesized that while the ELTD1 protein upregulation in Weri-Rb-1 cells could contribute to the adhesive properties that characterize these less invasive Rb cells, the ELTD1 protein expression and distribution in the Y79 cells could contribute to the invasive ability of these cells as they display lower adhesive characteristics. Therefore, after silencing of *ELTD1* in these Rb cells, we measured their migration using the Boyden Chamber system. The cells were added to the upper chamber with serum-free RPMI media; to stimulate migration of the cells through basement membrane, the lower chamber contained complete media to more closely mimic in vivo physiology. As a control, untransfected cells were also evaluated to ensure proper quantification of migrating Rb cells (Fig. 4, *right inserts*; white arrowheads). Our results demonstrated a significant reduction in migration in Y79 cells compared to Weri-Rb-1 cells (Fig. 4a-c, g-i; m; controls, untransfected: Y79 versus Weri-Rb-1: ****p < 0.0001; *N* = 4). This result extended to Y79 cells that were treated with the *siELTD1*, illustrating a significant decrease in migration when compared to untreated Y79 cells (Fig. 4a-c; d-f; m; Y79 controls, untransfected versus Y79 siELTD1: ***p < 0.001; *N* = 4). Quantitative analysis demonstrated that there was no statistical difference between *siELTD1* transfected Weri-Rb-1 cells and untransfected Weri-Rb-1 cells (Fig. 4g-i; j-l; m; *N* = 4). Next, we investigated the role of ELTD1 in cellular viability. As shown in Fig. 4n, there was no significant difference in cellular viability between treated and non-treated cells (Fig. 4n; *N* = 3). These findings support a role for ELTD1 in Rb tumor cell migration, but not cellular viability, in vitro.

### Silencing of *GPR125* did not decrease migration or viability of Rb cells

**Fig. 5:**
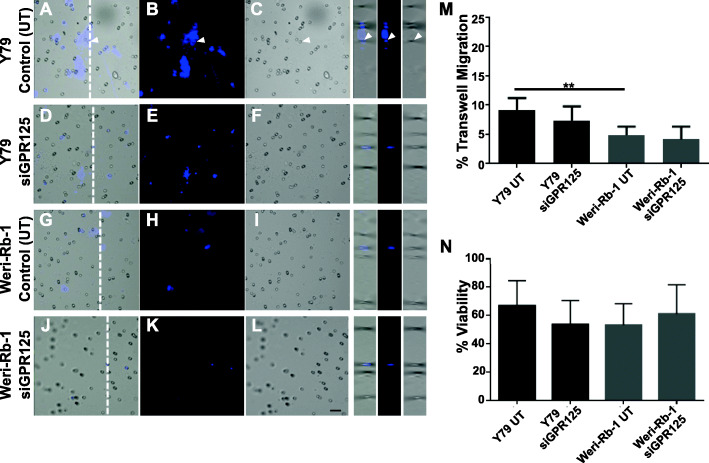
GPR125 does not play a role in Rb cell migration. **a-l** Subsequent to *GPR125* silencing, control untransfected (UT) cells (Y79: A-C; Weri-Rb-1: G-I), siGPR125 transfected Y79 (D-F) and *siGPR125* transfected Weri-Rb-1 cells (J-L) were we applied in serum free media in an upper chamber of an 8 um polycarbonate membrane. Cells were left incubated for 6 h to enable migration as stimulated by the supplemented media in the lower chamber. Migrating cells were fixed and stained with Hoechst dye for nuclear stain to ensure that quantification of blue migrating cells overlapped with the membrane’s pores (white arrowhead). **m** Quantitative analysis show a significant increase in migration of UT Y79 cells compared to UT Weri-Rb-1 cells, as expected. However, no reduction in migration was observed in the Rb cells after GPR125 silencing. **n**. Silencing of GPR125 did not have an effect in Rb cell viability. Ordinary One-Way ANOVA followed by Tukey’s multiple comparison test (M and N) was used to compare between columns. Scale bar for panels A-L is 10 μm. Error bars, SEM. Significance was expressed as **p < 0.01

To investigate the role of the adhesion GPR125 receptor in Rb, we performed similar experiments to those conducted with ELTD1 as described in the section above. Briefly, Rb cell lines were transfected with *siGPR125*. We hypothesized that, given the lack of *GPR125* RNA and protein expression difference between Rb tumors and fetal retinas (Fig. 1d and Fig. 2d, respectively), yet its protein overexpression in both, Weri-Rb-1 and Y79 cells, *siGPR125* could affect Rb cell migration or viability. As expected, there were differences in migratory activity between the Y79 and the Weri-Rb-1 cells (Fig. 5 a-c, g-i; m; controls, untransfected: Y79 versus Weri-Rb-1: **p < 0.01; *N* = 6). However, knockdown of *GPR125* did not have any effect on the migration (Fig. 5m; *N* = 6) of Y79 and Weri-Rb-1 cells when compared to their silenced counterparts. Cellular viability was not affected by *GPR125* gene knockdown in any of the Rb cell lines (Fig. 5n; *N* = 6).

### In vivo silencing of ELTD1 reduced optic nerve invasion in an orthotopic xenograft RB mouse model

**Fig. 6:**
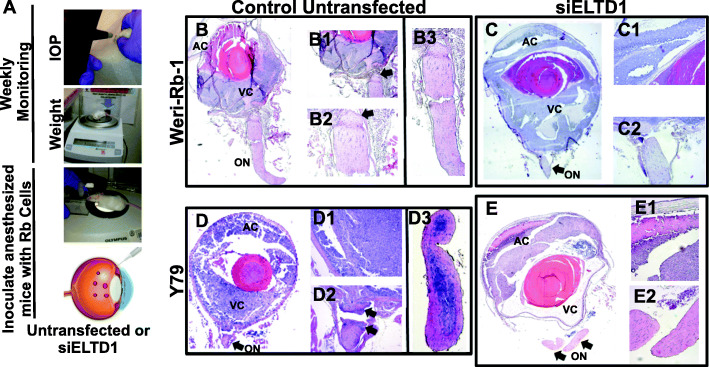
Inhibition of ELTD1 reduced optic nerve invasion in the aggressive Rb phenotypic cell line. **A** Schematic images illustrate procedure prior to Rb cell inoculation. Co-immunocompromised mice were weighted and intraocular pressure (IOP) was measured prior to and after Rb cell inoculation (controls and Y79 transfected with *siELTD1*). After mice reached an IOP ≥15 mmHg, closely near 2 months’ post-inoculation, mice were enucleated, and eyes for removed for histological analysis. **B, B1-B3** Controls untransfected Weri-Rb-1 cells show intraocular invasion, without extraocular involvement. **C, C1-C2** Eyes inoculated with transfected *siELTD1* Weri-Rb-1 cells displayed similar invasion patterns as their counterpart control conditions (B). **D, D1-D3** Controls untransfected Y79 cells show intraocular invasion, with extraocular optic nerve involvement. **E, E1-E3** Eyes inoculated with transfected *siELTD1* Y79 cells displayed substantial reduction in optic nerve invasion. Objective 4x was used for images B, B1, C, D and E while 10x was used for images B2-B3, C1-C2, D1-D3 and E1-E2. AC: Anterior Chamber; ON: Optic Nerve; VC: Vitreous Cavity

To test the hypothesis that ELTD1 influences Rb invasion from intraocular regions to the optic nerve, we developed an orthotopic xenograft Rb mouse model [5, 25, 41]. As demonstrated in Fig. 6A, anesthetized control mice (*N* = 5; inoculated with untransfected Y79 cells) as well as experimental mice (*N* = 7; inoculated with Y79 cells that were previously transfected with *siELTD1*), had their intraocular pressure (IOP) and weight measured prior to inoculation, and weekly, after cellular engraftment. A total of 50,000 cells in a 5 μL final volume were inoculated intravitreously in each eye of 8-weeks old male ICR-SCID mice. Tumors were visible after 2 weeks, as signs of anterior chamber invasion became obvious. Tumor growth was monitored for 2 months, after which orthotopic xenografts were euthanized and eyes were dissected for histopathological analysis, as before [5]. All eyes injected with untransfected Y79 cells demonstrated rapid tumor growth (*n* = 5 animals), while the *siELTD1*-Y79 (*n* = 7) inoculated eyes did not (data not shown). As expected, due to its less aggressive phenotype, untransfected Weri-Rb-1 (*n* = 5) and *siELTD1*-Weri-Rb-1 (*n* = 7) inoculated eyes, did not show rapid tumor growth. Hematoxylin and eosin (H&E) staining of the eyes demonstrated the dissemination of Y79 cells to other areas including the ON (Fig. 5d, D1–3), while Were-Rb-1 cells only invaded intraocular tissues (Fig. 5b, B1–3). Consistent with previous studies from members of our team and others [4, 17], Y79 cells exhibited aggressive invasion to the anterior chamber, the retina, the subretinal space, the choroid, and the optic nerve and Weri-Rb-1 limited their invasive capacity to intraocular regions [8, 17]. Silencing of *ELTD1* significantly reduced Y79 invasion to the optic nerve (Fig. 5e and E1-E2), while not displaying any change in the transfected non-metastatic Weri-Rb-1 cell (Fig. 5c and C1-C2).

## Discussion

Adhesion-GPCR are orphan receptors that play critical physiological and pathological roles. In cancer, their aberrant expression strongly contributes to disease development and progression. However, the expression profile, cellular and molecular roles of these adhesion GPCRs have not been investigated within the context of Rb. The work presented herein provides the first report of the RNA expression signature of adhesion-GPCRs, with a focus on ELTD1 and GPR125 functions in this intraocular pediatric malignancy.

ELTD1 and GPR125 are adhesion-GPCRs expressed in different tumors and have been linked to tumor progression [3, 13, 42]. Consistent with other tumors, Rb expresses *ELTD1* and *GPR125*; however, *ELTD1* RNA and protein were upregulated in Rb tumors when compared to fetal retina controls (Figs. 1 and 2). At the cellular level, ELTD1 was enriched in the less invasive Weri-Rb-1 cells where it highly distributed as punctate clusters in regions of cell-to-cell contact. In the more invasive Y79 cells, ELTD1 punctuate clusters are less pronounced and are distributed in cytoplasmic and nuclear regions. GPR125 was less intense and more diffused in nucleo-cytoplasmic regions of Weri-Rb-1 cells while in the Y79 cells, the protein distributed in a more polarized manner. The nucleo-cytoplasmic ELTD1 and GPR125 distribution pattern observed in Rb cells has been described for other proteins that shuttle between the nucleus and cytoplasm relaying information between these two cellular sites and zones of cell adhesion to extracellular matrix [15, 28]. Considering the unique structure of adhesion-GPCRs (e.g., long N-terminal with domains vital for adhesion), our results suggest that ELTD1 and GPR125 are distributed in this characteristic manner within Rb cells, and, thus, may represent a possible mechanism for transcellular coordination and communication, as well as for cell-to-cell and cell to matrix adhesion [15, 28].

ELTD1 has been associated with the development and progression of tumors given its role in cancer progression, including angiogenesis [3, 22]. Recent work from Kann and colleagues showed that silencing of *ELTD1* dramatically reduced invasiveness of hepatocellular carcinoma cells [16], confirming prior studies connecting ELTD1 to metastasis [42]. Furthermore, ELTD1 has been found to associate with poor prognosis in colorectal cancer [1]. Meanwhile, the role(s) of GPR125 in cancer remains poorly understood. Fu and colleagues suggested an oncogenic role in myeloid sarcoma [13], while others showed it works as a tumor suppressor in colorectal cancer by activation of the Wnt/β-catenin signaling pathway [38].

Our results showed that silencing of *ELTD1* decreased in vitro migration and in vivo invasion of the Y79 cells without affecting viability. Considering that ELTD1 protein was not upregulated in the Y79 cells, it is possible that these effects are affecting ELTD1 targets, which influence cell proliferation, differentiation, migration and apoptosis, and have been found to be altered in Rb [7, 18, 30].

Silencing of *GPR125* in Weri-Rb-1 or Y79 cells did not affect cell migration or viability. From its upregulation in Rb cells and polarized protein distribution in the Y79 cells, we hypothesized its knockdown would affect Rb cell migration. However, our results suggest that GPR125 could be contributing to other tumor-related processes that were beyond the scope of this manuscript, such as in Rb cell proliferation, cell death resistance, replicative potential, among others.

The variability in protein expression profile between Rb cell lines and primary tumors suggests different tumor microenvironmental conditions that may or may not be captured in vitro. The level of heterogeneity found in our studies highlights the importance of using distinct cell line models that reflect the complexity and heterogeneity characteristic of Rb tumors [8, 17].

Remaining questions that should be further examined include: (1) an investigation of the association between *ELTD1* and *GPR125* expression from the primary tumor throughout Rb development and progression as tumor metastasizes; (2) identification of ELTD1-associated mechanisms contributing to regulating Rb migration and invasion and, (3) examination of ELTD1’s role in Rb angiogenesis. Taken together, our in vitro, ex vivo and in vivo study reveal a novel biological platform that must be further explored as a potential personalized targeted therapy to better manage and treat metastatic Rb.

## Conclusion

This work reports differences in the expression and functional roles of two adhesion-GPCRs, ELTD1 and GPR125. We identified ELTD1, but not GPR125, to be overexpressed in primary Rb tumors and demonstrated that ELTD1 disruption reduces Y79 cell migration in vitro and metastasis in vivo without affecting cell viability. These results confirm a specific role for ELTD1 in migration and invasion and represent the first step towards a better comprehension of the expression profile and biological roles of adhesion-GPCRs in Rb with the hope of investigating and identifying new therapeutic avenues against metastatic Rb.

## Supplementary Information

**Additional file 1: Supplementary Table 1.** RNA expression microarray illustrates expression profile of adhesion-GPCRs that were significantly up- or downregulated in Rb pediatric tumors (*N* = 64) compared to fetal retina controls (*N* = 11), and those that did not display significant differences.

**Additional file 2: Supplementary Figure 1.** Full-length Western blot Shown in Fig. 2. ***A.*** *hela* cell, not human brain, lysates show immunopositivity against the anti-ELTD1 antibody shown by the 77kDA band. GAPDH, a 37kDA protein, was used as loading control. **B.** Full length image measuring immunopositivity against ELTD1 in fetal retina (Fet Ret), Rb cells and Rb tumors. GAPDH was used as loading control. **C.** HeLa cell, not Jurkat cells, lysates show immunopositivity against the anti-GPR125 antibody shown by the 146kDA band. GAPDH was used as loading control. **D.** Full length image measuring immunopositivity against GPR125 in Fet Ret, Rb cells and Rb tumors. GAPDH was used as loading control. For all full-length blots, dotted line boxes around sample lanes indicate the area of the membrane that was cropped in Fig. 2.

## Data Availability

The datasets used and/or analyzed during this study are available from the corresponding author on reasonable request. Information on Y79 and Weri-Rb-1 Rb cells are available in www.ncbi.nlm.nih.gov/biosample and in www.atcc.org.

## Abbreviations

Rb: Retinoblastoma
ON: Optic nerve
adhesion-GPCRs: Adhesion G protein-coupled receptors
ELTD1: Epidermal growth factor; latrophilin and seven transmembrane domain containing 1
GPR125: G-protein receptor 125
CNS: Central nervous system
RPMI: Roswell Park Memorial Institute
FBS: Fetal Bovine Serum
CSTN: Childhood Solid Tumor Network
SJCRH: St Jude Children’s Research Hospital
DMEM: Dulbecco’s modified Eagle’s medium
RT-PCR: *Reverse transcription polymerase chain reaction*
*RNA*: *Ribonucleic Acid*
cDNA: Complementary DNA
HPRT1: Hypoxanthine Phosphoribosyltransferase 1
SEM: Standard Error of the Mean
UPR: University of Puerto Rico
IRB: Institutional Review Board
IACUC: American Association for Laboratory Animal Science
PR: Puerto Rico
RIPA: Radioimmunoprecipitation assay buffer
PVDF: Polyvinylidene fluoride
BM: Basement membrane
TBS-T: Tris buffered saline with Tween 20
TBS: Tris buffered saline
BSA: Bovine serum albumin
HRP: Horseradish peroxidase
SDS: Sodium dodecyl sulfate
PFA: Paraformaldehyde
PBS: Phosphate Buffered Saline
DAPI: 4′,6-Diamidino-2-Phenylindole dihydrochloride
siRNA: Small interfering RNA
IOP: Intraocular pressure
mRNA: Messenger RNA
H&E: Hematoxylin and eosin
GBM: Glioblastoma multiforme
HCC: Hepatocellular carcinoma
PCP: Planar cell polarity
Dvl: Dishevelled proteins
