# Supplementary material for: Adhesion G protein-coupled receptor, ELTD1, is a potential therapeutic target for retinoblastoma migration and invasion
Source: BMC Cancer. 2021 Jan 11;21:53. doi: 10.1186/s12885-020-07768-3 (PMC7802354; doi:10.1186/s12885-020-07768-3)
Supplement: Supplementary file 1 — Additional file 1: Supplementary Table 1. RNA expression microarray illustrates expression profile of adhesion-GPCRs that were significantly up- or downregulated in Rb pediatric tumors (N = 64) compared to fetal retina controls (N = 11), and those that did not display significant differences. [file 12885_2020_7768_MOESM1_ESM.pdf]

Additional File 1: Supplementary Table 1. Microarray analysis show expression of adhesion-GPCRs in retinoblastoma pediatric tumors compared to fetal retinae

Adhesion-GPCRs Subfamily	Gene	F Value	Prob > F	Up- or Downregulation in Retinoblastoma Tumors
I	LPHN1	0.6	0.4408	ND
I	LPHN2	27.08	<0.0001	Downregulated
I	LPHN3	26.83	<0.0001	Downregulated
I	ELTD1	20.25	<0.0001	Upregulated
II	EMR1	25.24	<0.0001	Upregulated
II	EMR2	9.55	0.0028	Upregulated
II	EMR3	2.41	0.1251	ND
II	EMR4	TBD	TBD	TBD
II	CD97	4.58	0.0356	Upregulated
III	GPR123	36.24	<0.0001	Upregulated
III	GPR124	24.89	<0.0001	Downregulated
III	GPR125	1.89	0.1731	ND
IV	CELSR1	30.62	<0.0001	Upregulated
IV	CELSR2	52.98	<0.0001	Downregulated
IV	CELSR3	0.00	0.9864	ND
V	GPR133	4.55	0.0364	Downregulated
V	GPR144	0.36	0.5497	ND
VI	GPR110	1.62	0.2066	ND
VI	GPR111	1.54	0.2193	ND
VI	GPR113	10.08	0.0022	Upregulated
VI	GPR115	2.87	0.0944	ND
VI	GPR116	14.53	0.0003	Downregulated
VII	BAI1	0.97	0.329	ND
VII	BAI2	0.15	0.7037	ND
VII	BAI3	1.75	0.1902	ND
VIII	GPR56	3.95	0.0507	Downregulated
VIII	GPR64	0.15	0.7033	ND
VIII	GPR97	3.74	0.0571	Upregulated
VIII	GPR112	0.92	0.3394	ND
VIII	GPR114	3.63	0.0608	ND
VIII	GPR126	3.72	0.0577	ND
VIII	GPR128	1.90	0.1722	ND
IX	VLGR1	TBD	TBD	TBD

Additional File 1: Supplementary Table 1. Expression data from the Childhood Solid Tumor Network at St Jude Children's Research Hospital (Stewart et al., 2015). This dataset of genes was analyzed from 64 human Rb tumors compared to 11 fetal retinae using the JMP v12.2 Software (*www.jmp.com/en_us/software/jmp.html*) to conduct a

hierarchical cluster analysis. Upregulated adhesion-GPCRs include: ELTD1, EMR1, EMR2, CD97, GPR123, CELSR1, GPR113 and GPR97. Downregulated adhesion-GPCRs include: LPHN2, LPHN3, GPR124, CELSR2, GPR133, GPR116 and GPR56. Adhesion-GPCRs that did not display any difference between retinoblastoma tumors and fetal retinas include: LPHN1, EMR3, GPR125, CELSR3, GPR144, GPR110, GPR111, GPR115, BAI1, BAI2, BAI3, GPR64, GPR112, GPR114, GPR126 and GPR128. EMR4 and VLGR1 remain to be examined. TBD: To be determined.
