# Supplementary figures and images for: Adhesion G protein-coupled receptor, ELTD1, is a potential therapeutic target for retinoblastoma migration and invasion
Source: BMC Cancer. 2021 Jan 11;21:53. doi: 10.1186/s12885-020-07768-3 (PMC7802354; doi:10.1186/s12885-020-07768-3)

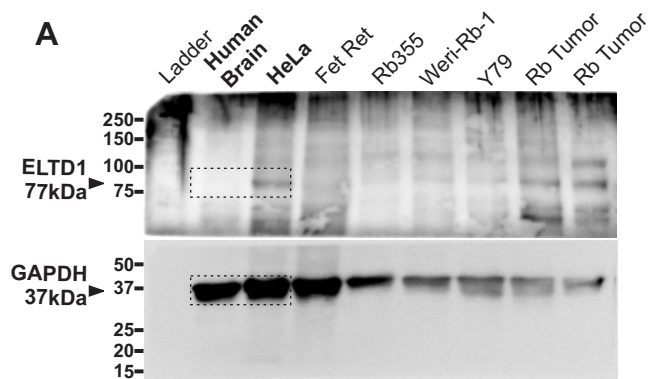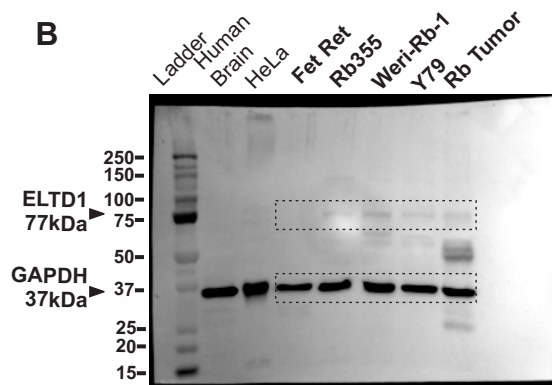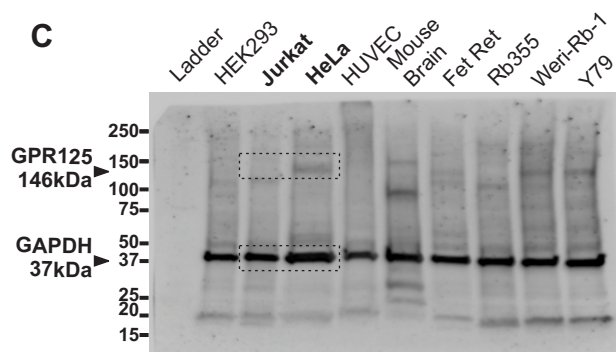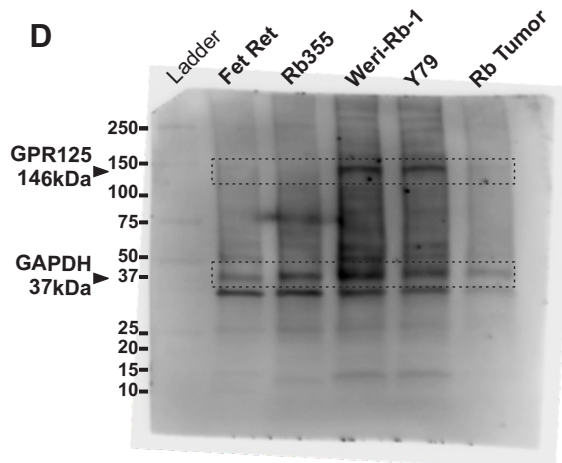

Supplement: Supplementary file 2 — Additional file 2: Supplementary Figure 1. Full-length Western blot Shown in Fig. 2. A. hela cell, not human brain, lysates show immunopositivity against the anti-ELTD1 antibody shown by the 77kDA band. GAPDH, a 37kDA protein, was used as loading control. B. Full length image measuring immunopositivity against ELTD1 in fetal retina (Fet Ret), Rb cells and Rb tumors. GAPDH was used as loading control. C. HeLa cell, not Jurkat cells, lysates show immunopositivity against the anti-GPR125 antibody shown by the 146kDA band. GAPDH was used as loading control. D. Full length image measuring immunopositivity against GPR125 in Fet Ret, Rb cells and Rb tumors. GAPDH was used as loading control. For all full-length blots, dotted line boxes around sample lanes indicate the area of the membrane that was cropped in Fig. 2. [file 12885_2020_7768_MOESM2_ESM.pdf]
